# Comparison of plasma levobupivacaine concentrations with and without epinephrine following erector spinae plane block for breast cancer surgery: a randomized controlled trial

**DOI:** 10.1186/s12871-022-01632-6

**Published:** 2022-03-29

**Authors:** Hiroe Shigeta, Rie Yasumura, Yoshifumi Kotake

**Affiliations:** 1 Department of Anesthesiology, Toho University Graduate School of Medicine, Tokyo, Japan; 2 Department of Anesthesia, National Hospital Organization, Tokyo Medical Center, Tokyo, Japan; 3 Department of Anesthesiology, School of Medicine, Toho University, Tokyo, Japan

**Keywords:** Erector spinae plane block, Plasma levobupivacaine concentrations, Epinephrine

## Abstract

**Background:**

The erector spinae plane (ESP) block requires a large volume of local anesthetic to provide effective analgesia, which has the potential to cause local anesthetic systemic toxicity (LAST). Adjunctive epinephrine slows the entry of local anesthetic into the plasma and decreases its toxic effect on vulnerable tissues. We compared plasma levobupivacaine concentrations with and without epinephrine after ESP blocks for breast cancer surgery.

**Methods:**

In this prospective, double-blinded, randomized controlled trial, 35 patients who underwent elective unilateral partial mastectomy with sentinel lymph node biopsy were enrolled. The patients were randomized to group L (ESP block with 2 mg/kg levobupivacaine) or LE (ESP block with 2 mg/kg levobupivacaine and 5 μg/mL epinephrine). Blood samples were obtained at 2.5, 5, 7.5, 10, 12.5, 15, 30, 60, and 120 min after the ESP block, and plasma concentrations of levobupivacaine were compared.

**Results:**

Twenty-nine patients were included in the analysis. The maximum plasma concentration (C_max_) and the time to maximum concentration (T_max_) were, respectively, 1.24 μg/mL and 6.0 min in group L and 0.62 μg/mL and 7.2 min in group LE. The two groups showed no significant differences in the numerical rating scale scores immediately after extubation and 5 and 9 h after the ESP block, or in the interval from the ESP block to the first rescue analgesia. No patient developed symptoms suggestive of LAST.

**Conclusions:**

A single bolus of 2 mg/kg levobupivacaine in the ESP block resulted in a short T_max_ with high C_max_. Adding epinephrine to levobupivacaine decreased the C_max_ and delayed the T_max_ after ESP blocks but had no effect on postoperative analgesia.

**Trial registration:**

UMIN Clinical Trials Registry, UMIN000034479. The trial was retrospectively registered on October 13, 2018.

**Supplementary Information:**

The online version contains supplementary material available at 10.1186/s12871-022-01632-6.

## Background

Persistent pain is a frequent and common problem after breast cancer surgery and is associated with reduced quality of life and functional impairments [1]. Approximately 37% of patients experience this complication after breast cancer surgery [2]. Acute postoperative pain is considered to be a risk factor for persistent pain after breast cancer surgery [3], and many previous studies have attempted to evaluate approaches to relieve such postoperative pain. In particular, to provide sufficient perioperative analgesia, peripheral nerve blocks, including thoracic paravertebral block (TPVB), pectoral nerve block I and II, and serratus plane block, are frequently used in breast surgery. TPVB is the most widely used procedure for thoracic analgesia; however, it is associated with the risk of vascular puncture, hypotension, pleural puncture, and pneumothorax [4, 5], necessitating the development of alternative techniques [6]. The erector spinae plane (ESP) block, one of the “paravertebral by proxy” blocks, was first reported in 2016 for thoracic neuropathic pain [7], and many studies have reported the effectiveness of ESP blocks for breast cancer surgery [8, 9].

Local anesthetic systemic toxicity (LAST) is a critical adverse event that may occur after peripheral nerve blocks. The peak plasma concentration of local anesthetics and the time to reach the peak concentration are defined by the rate of systemic absorption, which is related to the vascular supply and the contact area between the local anesthetic and the vascular bed of the injected plane. The erector spinae muscles of the thorax are supplied by the posterior and superior intercostal arteries and are considered to have a rich vascular supply [10]. To provide adequate analgesia, the ESP block requires a large volume of local anesthetics, which spreads over a large surface area of the erector spinae muscle. Therefore, the peak plasma concentration of local anesthetics potentially increases after ESP block and is considered a risk factor for LAST [11, 12]. However, there is little information related to levobupivacaine pharmacokinetics after ESP block.

Addition of epinephrine is one of the methods to mitigate the systemic uptake of local anesthetics: adjunctive epinephrine slows the entry of the local anesthetic into the plasma and reduces the peak plasma concentration [13]. However, the addition of epinephrine did not reduce the peak plasma ropivacaine concentration in the rectus sheath block [14]. Thus, the effect of adjunctive epinephrine on the pharmacokinetics of local anesthetics has been reported to depend on the properties of the local anesthetic and the vascular density of the injected site [14]. In this regard, determination of the effect of adjunctive epinephrine on the pharmacokinetics of levobupivacaine in the ESP block may facilitate the prevention of LAST.

Currently, there are no studies evaluating the effect of adjunctive epinephrine on levobupivacaine and its pharmacokinetics in ESP blocks. Therefore, we aimed to investigate the pharmacokinetics of levobupivacaine with and without epinephrine during ESP blocks.

## Methods

This double-blind randomized controlled trial was approved by the ethics committee of the National Hospital Organization Tokyo Medical Center (R18-006) and registered in the UMIN Clinical Trials Registry (UMIN000034479: 13/10/2018). Written informed consent was obtained from all participants.

### Patients and randomization

Women aged 20 to 74 years with ASA physical status classification I–III who underwent elective unilateral partial mastectomy with sentinel lymph node biopsy (SLNB) under general anesthesia between June 2018 and April 2021 were enrolled in this study. Patients with an allergy or a contraindication to any of the medications used in the study, coagulopathy, or a history of renal or hepatic dysfunction were excluded from the study.

All patients were randomly assigned to one of two groups in a 1: 1 ratio: patients in group L received an ESP block with levobupivacaine alone and those in group LE received an ESP block with levobupivacaine combined with epinephrine. Block randomization was achieved using a computer-generated randomization list created by an independent anesthesiologist. Before induction of anesthesia, a nurse practitioner in the central clinical facility of our institution who was not involved in the study prepared the study drugs (2 mg/kg levobupivacaine diluted with 0.9% saline to a volume of 30 mL with or without 5 μg/mL epinephrine) according to the randomization list. Masking was achieved using apparently identical 30-mL syringes. The study drug was given to the anesthesiologist before performing the ESP block.

### Block technique

An experienced anesthesiologist performed all the ESP blocks under ultrasound guidance by using EDGE (FUJIFILM SonoSite, Inc., Bothell, WA, USA) with a 6–15-MHz linear probe. After induction of general anesthesia, patients were placed in the left lateral decubitus position, and an ESP block was administered at the T4 thoracic vertebral level. A 22-G 80-mm disposable nerve blockade needle (Uniever; Unisis, Tokyo, Japan) was inserted caudocranially using the in-plane technique, and the study drug was injected into the fascial plane between the erector spinae muscle and the transverse process after confirming that blood was not aspirated.

### Intraoperative and postoperative management

Unpremedicated patients were transferred to the operating room, where standard monitoring including pulse oximetry, non-invasive blood pressure measurement, electrocardiography, and skin surface temperature and bispectral index (BIS; Covidien/Medtronic, USA) measurements was applied. After insertion of an intravenous catheter, anesthesia was induced with 2–3 mg/kg propofol and 2 μg/kg fentanyl. The trachea was intubated after 0.6 mg/kg rocuronium administration, and mechanical ventilation was initiated. Anesthesia was maintained with propofol and remifentanil to ensure that the BIS value remained between 40 and 60 and mean blood pressure was within 20% of baseline measurements. Additional doses of fentanyl were administered such that the serum concentration calculated by the Shafer model was 1.0 ng/mL at the end of the surgery. Local wound infiltration was not permitted at any time of the study.

Postoperative pain was assessed soon after extubation and 5 h and 9 h after the blockade by using a 11-point numerical rating scale (NRS), where 0 indicated no pain and 10 indicated the worst pain. Postoperative pain was assessed by the members of acute pain service team, and non-steroidal anti-inflammatory drugs or acetaminophen was administered if required.

### Plasma levobupivacaine measurements

All patients received a dedicated intravenous line contralateral to the fluid line for blood sampling. Venous blood samples of 2 mL each were obtained at 2.5, 5, 7.5, 10, 12.5, 15, 30, 60, and 120 min after completion of the blockade. Each blood sample was collected in a tube containing heparin and immediately placed on ice. Plasma was separated by centrifugation of blood samples at 1,500 × *g* for 10 min and stored at -20 °C until subsequent analyses. Plasma levobupivacaine concentrations were measured using high-performance liquid chromatography (Accela; Thermo Fisher Scientific Co., Ltd., Kanagawa, Japan) and liquid chromatography-mass spectrometry with a triple-stage quadrupole mass spectrometer (TSQ Quantum Ultra; Thermo Fisher Scientific Co., Ltd.) or high-performance liquid chromatography (Vanquish Flex; Thermo Fisher Scientific K.K., Tokyo, Japan) and liquid chromatography-mass spectrometry with a triple-stage quadrupole mass spectrometer (TSQ Altis; Thermo Fisher Scientific K.K.).

### Outcomes

The primary outcome was plasma concentrations of levobupivacaine with and without epinephrine after ESP blocks. The secondary outcomes were NRS scores soon after extubation and 5 h and 9 h after the blockade and the interval between the completion of the block and the first analgesic request.

### Statistical analysis

Plasma levobupivacaine concentrations within and between groups were analyzed using two-way repeated-measures analysis of variance (two-way repeated ANOVA) with Bonferroni post-hoc comparisons. The interval between the completion of the block and the first analgesic request was analyzed using the Kaplan–Meier survival analysis with the log-rank test. Baseline and surgical characteristics were compared using the t-test or the Mann–Whitney U-test. The maximum plasma concentration (C_max_) and time to maximum concentration (T_max_) were calculated using nonlinear regression analysis in a two-compartment pharmacokinetic model. Goodness-of-fit was assessed using the coefficient of determination (R^2^). Statistical significance was set at *p* < 0.05. Data were analyzed using GraphPad Prism version 8.0 (GraphPad Software, San Diego, CA, USA).

### Sample size calculation

The sample size calculation was performed using G*Power 3 with no pilot study data. Assuming a dropout rate of 10%, we determined that a sample size of 34 patients was needed to perform two-way repeated ANOVA, with a two-sided alpha of 0.05, power of 0.8, and effect size of 0.25.

## Results

**Fig. 1:**
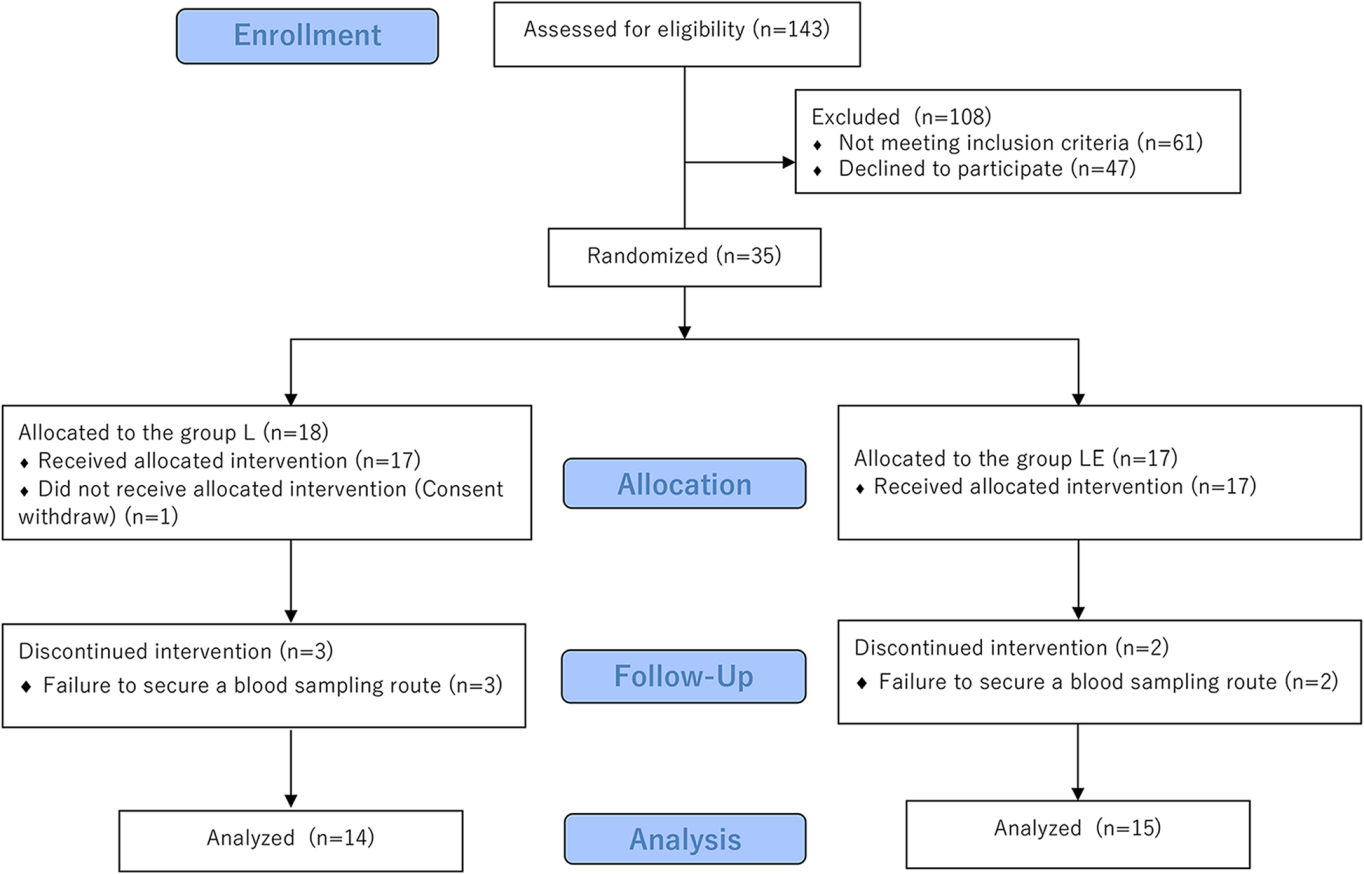
Consolidated Standards of Reporting Trials flow diagram showing participant recruitment. L, levobupivacaine; LE, levobupivacaine + epinephrine

One hundred and forty-three patients scheduled for elective unilateral partial mastectomy with SLNB between June 2018 and April 2021 were assessed for eligibility, and a total of 35 patients were enrolled and randomized to either group L or group LE. Among them, 34 patients (17 per group) received the allocated intervention. After excluding five patients owing to failure to secure a blood sampling route, 29 were included in the analysis: 14 were allocated to group L, and 15 were allocated to group LE (Fig. 1). The two groups were comparable in terms of patient and surgical characteristics (Table 1).

**Table 1:** Patient and surgical characteristics

	Group L	Group LE
Sample size, n	14	15
Patient characteristics		
Mean age (SD; years)	52.5 (8.0)	55.4 (9.8)
Mean weight (SD; kg)	56.2 (6.9)	55.3 (4.5)
Mean BMI (SD; kg/m^2^)	22.9 (3.0)	22.1 (2.2)
ASA classification, n (%)		
I	9 (64.3)	8 (53.3)
II	5 (35.7)	7 (46.7)
ALND, n (%)		
+	2 (14.3)	0 (0.0)
–	12 (85.7)	15 (100.0)
Surgical characteristics		
Mean surgical time (SD; min)	107.4 (20.5)	98.1 (22.0)
Mean anesthesia time (SD; min)	153.6 (22.5)	145.7 (22.1)
Mean levobupivacaine dose (SD; mg)	112.0 (14.2)	110.7 (10.1)

*SD* standard deviation, *BMI* body mass index, *ASA* American Society of Anesthesiologists, *ALND* axillary lymph node dissection

**Fig. 2:**
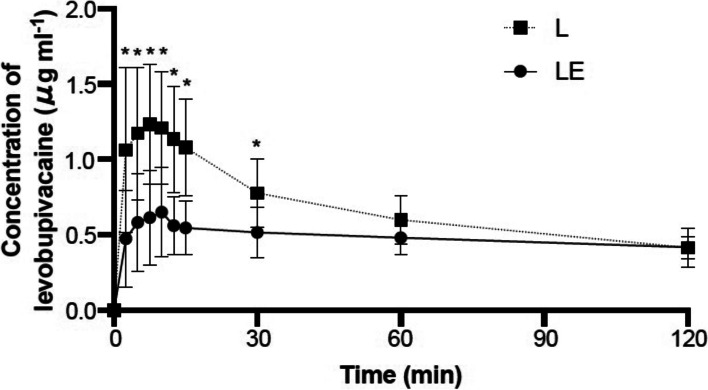
Mean time–concentration profile of plasma levobupivacaine. Data are expressed as mean ± SD. *p < 0.05. L, levobupivacaine; LE, levobupivacaine + epinephrine

The time course of plasma levobupivacaine concentrations with and without epinephrine is shown in Fig. 2. The mean (SD) peak concentration was 1.23 (0.39) μg/mL at 7.5 min after injection in group L, and 0.65 (0.30) μg/mL at 10 min after injection in group LE. The mean concentrations at 2.5, 5, 7.5, 10, 12.5, 15, and 30 min after the ESP block were significantly higher in group L. The highest individual plasma levobupivacaine concentration was 2.28 μg/mL, which was observed in a patient of group L.

Nonlinear regression analysis showed that the C_max_ and T_max_ were, respectively, 1.24 μg/mL and 6.0 min in group L and 0.62 μg/mL and 7.2 min in group LE. R^2^ was 0.60 and 0.39 in group L and LE, respectively. The two groups showed no significant differences in terms of intraoperative opioid consumption and postoperative pain scores (Table 2).

**Table 2:** Intraoperative opioid consumption and postoperative pain scores

	Group L	Group LE	*P* value *†
Sample size, n	14	15	
Intraoperative opioid consumption			
Mean fentanyl dose (SD; µg)	276.4 (41.7)	263.7 (41.9)	0.42
Mean remifentanil dose (SD; µg/kg/min)	0.11 (0.01)	0.11 (0.02)	0.76
Median postoperative NRS (IQR)			
Soon after extubation	0.0 (0.0–2.8)	0.0 (0.0–3.0)	0.45
5 h after the ESP block	3.0 (2.8–3.5)	4.0 (2.0–5.0)	0.65
9 h after the ESP block	2.0 (1.0–3.0)	2.0 (1.0–4.0)	0.60

^*^P value for comparison between groups L and LE. † The t-test was used to compare means and the Mann–Whitney U-test was used to compare medians. *SD* standard deviation, *NRS* numerical rating scale, *IQR* interquartile range, *ESP* erector spinae plane

**Fig. 3:**
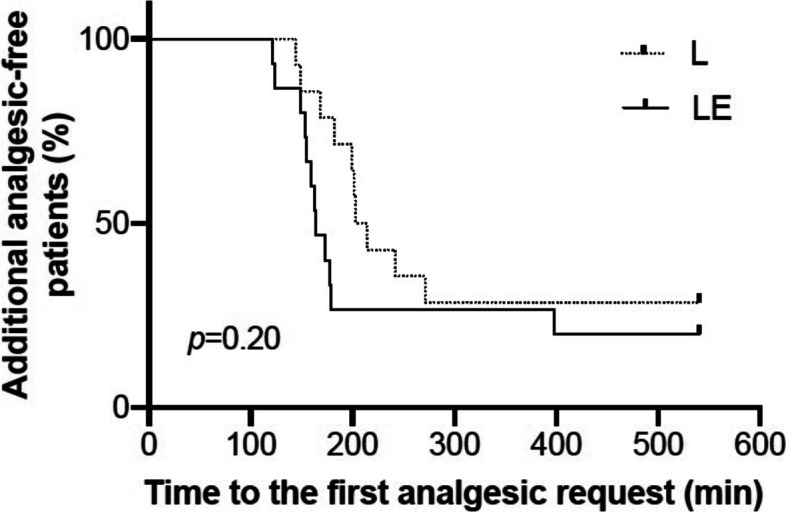
Kaplan–Meier curves for additional analgesic-free patients after erector spinae plane block for both groups. L, levobupivacaine; LE, levobupivacaine + epinephrine

Kaplan–Meier survival analysis showed no significant difference in the interval from the ESP block to the first analgesic request between groups L and LE (hazard ratio: 1.71; 95% confidence interval: 0.73–4.00; *p* = 0.20) (Fig. 3).

None of the patients developed symptoms suggestive of LAST or accidental intravascular administration of epinephrine.

## Discussion

To the best of our knowledge, this is the first study to prospectively investigate the plasma concentrations of levobupivacaine with and without epinephrine after ESP blocks in a double-blinded controlled setting. Since Forero et al. first described the ESP block in 2016 [7], this technique has attracted much interest among anesthesiologists. Most of the previous studies on ESP blocks have investigated their mechanisms of action and analgesic effect [8, 9], and only a few studies evaluated the pharmacokinetics of local anesthetics after the ESP block.

The peak plasma concentration of the local anesthetic after an ESP block is considered to potentially rise higher than that after a paravertebral block [11], since a large volume of local anesthetic is injected to the surface of a highly vascularized muscle [10]. In contrast, Taketa et al. [15] reported that the plasma concentrations of levobupivacaine after the ESP block were significantly lower than those after TPVB. They considered that the plasma concentration was kept low because the drug was injected into the loose connective tissue in the interfacial plane in the ESP block [16]. Notably, in the study by Taketa et al., levobupivacaine was administered through a catheter and then infused continuously, so the plasma concentration of levobupivacaine immediately after administration of the local anesthetic bolus was not measured. As shown above, the pharmacokinetics of levobupivacaine after the ESP block remain unknown.

The study by De Cassai et al. [12] showed that the mean T_max_ of lidocaine after bilateral ESP blocks in 10 patients was shorter than that in other studies investigating lidocaine pharmacokinetics. In the current study, the T_max_ of levobupivacaine in group L (6.0 min) was shorter than that for other peripheral nerve blocks or epidural anesthesia with levobupivacaine [17]: the short T_max_ in the ESP block suggested a high rate of absorption from the injection site.

The effect of adjunctive epinephrine on the plasma concentration of local anesthetics has been reported to depend on the vascular density or blood flow at the injected site [14]. In the ESP block, the local anesthetics are injected into a vascular-rich area; therefore, adjunctive epinephrine is expected to decrease the C_max_ and prolong the T_max_ after the block. In the current prospective randomized controlled trial, we revealed that the addition of 1:200,000 epinephrine to levobupivacaine (group LE) contributed to a 50% reduction in the C_max_ and delayed the T_max_.

LAST is a critical adverse event that may occur after a peripheral nerve block. Although the incidence of LAST associated with peripheral nerve blocks is decreasing owing to the use of ultrasound imaging, seizure or cardiac arrest still occurs at an estimated rate of 2.6/10,000 ultrasound-guided blocks [18]. Even the ESP block is no exception, and several reports have described the development of LAST after an ESP block [19–21]. Yawata et al. [19] performed bilateral lumbar ESP blocks at the L4 level by using 2.6 mg/kg of levobupivacaine, resulting in convulsions in a patient. Another study by Lee et al. [20] reported that a unilateral ESP block at the T6 level by using 1.3 mg/kg of lidocaine caused symptoms of a psychotic reaction. These studies suggest that an ESP block may produce LAST even if local anesthetics below the maximum recommended dose are administered.

Adjunctive epinephrine slows the entry of local anesthetic into the plasma, delays systemic uptake, and decreases the toxic effect on vulnerable tissues, such as the myocardium and central nervous system (CNS) [17]. The toxic effect of local anesthetics is determined by the peak plasma concentration, and the toxic threshold that causes CNS symptoms in healthy volunteers was reported to be equivalent to a venous levobupivacaine concentration of 2.62 μg/mL [22]. The current study demonstrated that C_max_ did not reach the toxic threshold of plasma levobupivacaine concentration regardless of the addition of epinephrine, and no symptoms were observed, indicating LAST. However, the individual highest plasma concentration was 2.28 μg/mL when epinephrine was absent. Whether a patient develops LAST is determined not only by a single threshold for plasma concentration but also by multiple factors, including age, comorbidities, and concurrent drug use [17]. Considering our result that C_max_ was reduced almost by half with adjunctive epinephrine, we recommend that epinephrine should be added to local anesthetics when performing ESP block for patients who are at risk of developing LAST (e.g., bilateral blockade, extremes of age, cardiac disease, and liver disease) [13].

In this study, the pain scores recorded soon after extubation and at 5 and 9 h after the blockade as well as the time to first rescue analgesia after the blockade were comparable between the groups. Most previous studies that compared the duration of blockade using levobupivacaine with and without epinephrine showed that the addition of epinephrine did not affect the duration of the blockade [23, 24]. In general terms, vasoconstrictors do not prolong the duration of blockade when added to long-acting local anesthetics such as ropivacaine and levobupivacaine [25], although the underlying mechanism remains unclear. Meanwhile, the putative mechanisms of action in fascial plane blocks, including ESP blocks, involve (1) local anesthetic spread to target nerves and surrounding tissues, and (2) systemic effect of absorbed local anesthetics [26]. With respect to the systemic effect of local anesthetics, several studies [27, 28] have reported the analgesic properties of intravenous lidocaine infusion and its therapeutic thresholds of plasma concentration. Long-acting local anesthetics are expected to have a similar systemic effect [26]. In the present study, we could not find a significant association between plasma levobupivacaine concentrations and postoperative pain, but the analysis of postoperative pain was underpowered.

In the current study, 5 out of 34 patients were excluded from the final analysis, with a higher dropout rate than initially expected. However, according to the post hoc power analysis, it revealed that 29 patients were enough for maintaining the power (which was still over 0.8).

This study had several limitations. First, the free fraction of plasma levobupivacaine, which is responsible for tissue toxicity, was not measured. Levobupivacaine is a highly protein-binding drug, but in conditions with low plasma protein concentrations, the proportion of unbound levobupivacaine is increased [17]. In our study, patients were relatively young, had fewer comorbidities, and did not develop symptoms suggestive of LAST. However, when performing the ESP block in patients with low protein levels, the addition of epinephrine to local anesthetics should be considered. Second, plasma levobupivacaine concentrations were measured using venous blood samples. To evaluate local anesthetic toxicity, arterial blood sampling is considered important because the arterial blood concentration of local anesthetic reflects the tissue concentration affected [29]. Considering the invasiveness of the surgery, we collected venous blood samples. Finally, the loss of sensibility was not assessed because the wounds were covered with a multi-layered compression bandage.

## Conclusions

In conclusion, a single bolus of 2 mg/kg levobupivacaine in the ESP block resulted in a short T_max_ with high C_max_. The addition of 1:200,000 epinephrine to levobupivacaine decreased the C_max_ and delayed the T_max_ after the ESP block, but had no effect on postoperative analgesia. When performing an ESP block, the use of adjunctive epinephrine with local anesthetics might be an option for patients who are at risk of developing LAST.

## Supplementary Information

**Additional file1:**

## Data Availability

All data generated or analysed during this study are included in this published article and its supplementary information files.

## Abbreviations

ANOVA: Analysis of variance
ASA: American Society of Anesthesiologists
C_max_: Maximum plasma concentration
CNS: Central nervous system
ESP: Erector spinae plane
LAST: Local anesthetic systemic toxicity
NRS: Numerical rating scale
SLNB: Sentinel lymph node biopsy
T_max_: Time to maximum concentration
TPVB: Thoracic paravertebral block
